# Supplementary material for: Self-supervised dynamic learning for long-term high-fidelity image transmission through unstabilized diffusive media
Source: Nat Commun. 2024 Feb 19;15:1498. doi: 10.1038/s41467-024-45745-7 (PMC10876540; doi:10.1038/s41467-024-45745-7)
Supplement: Supplementary file 1 — Supplementary Information [file 41467_2024_45745_MOESM1_ESM.pdf]

Supplementary of

Self-supervised dynamic learning for long-term high-fidelity image transmission through unstabilized diffusive media

Supplementary Information Table

**Supplementary Figure 1**	Schematic of a multimode fiber-based spatial transmission system
**Supplementary Figure 2**	Characterization of MMF transmission channel stability
**Supplementary Figure 3**	Characterization of abrupt distortion of MMF channel
**Supplementary Figure 4**	Schematic of the multi-timescale network architecture and training
**Supplementary Figure 5**	Comparison of multi-scale memory ensemble and long-term only dynamic learning network
**Supplementary Figure 6**	Comparison in reconstruction accuracy between MMDN and TM-based algorithms.
**Supplementary Figure 7**	Efficient pixel reassignment of the transmitted spatial patterns
**Supplementary Figure 8**	Transmission of incompressive encoded video via MMFs
**Supplementary Figure 9**	Comparison of MMDN and state-of-art approaches for spatial decoding in MMFs
**Supplementary Figure 10**	Summary of specifications of the MMFs
**Supplementary Figure 11**	Performance evaluation with respect to the number of training dataset
**Supplementary Figure 12**	Experimental comparison with SMF-based image transmission

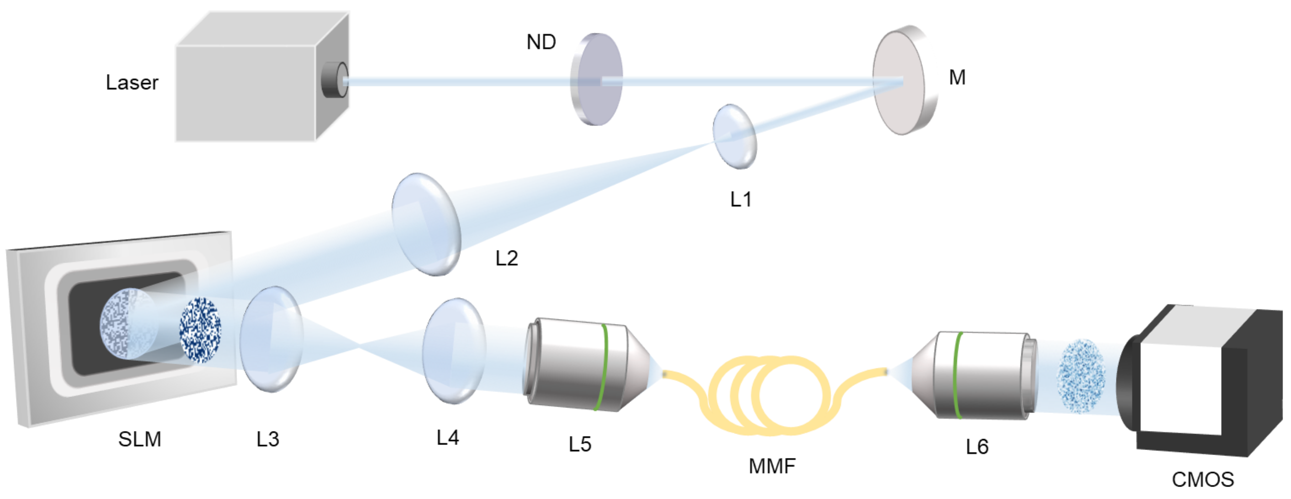

Supplementary Figure 1

Schematic of a multimode fiber-based spatial transmission system. The experimental setup of the amplitude-to-amplitude multimode fiber (MMF) spatial transmission system is illustrated. ND: neutral density filter, OD=1; L1, L2: aspherical lens, f=10mm, 100mm; L3, L4: aspherical lens, f=50mm, 50mm; L5, L6: objectives, Nikon, 20X, 0.25NA; SLM: spatial Light Modulator, V-7001.

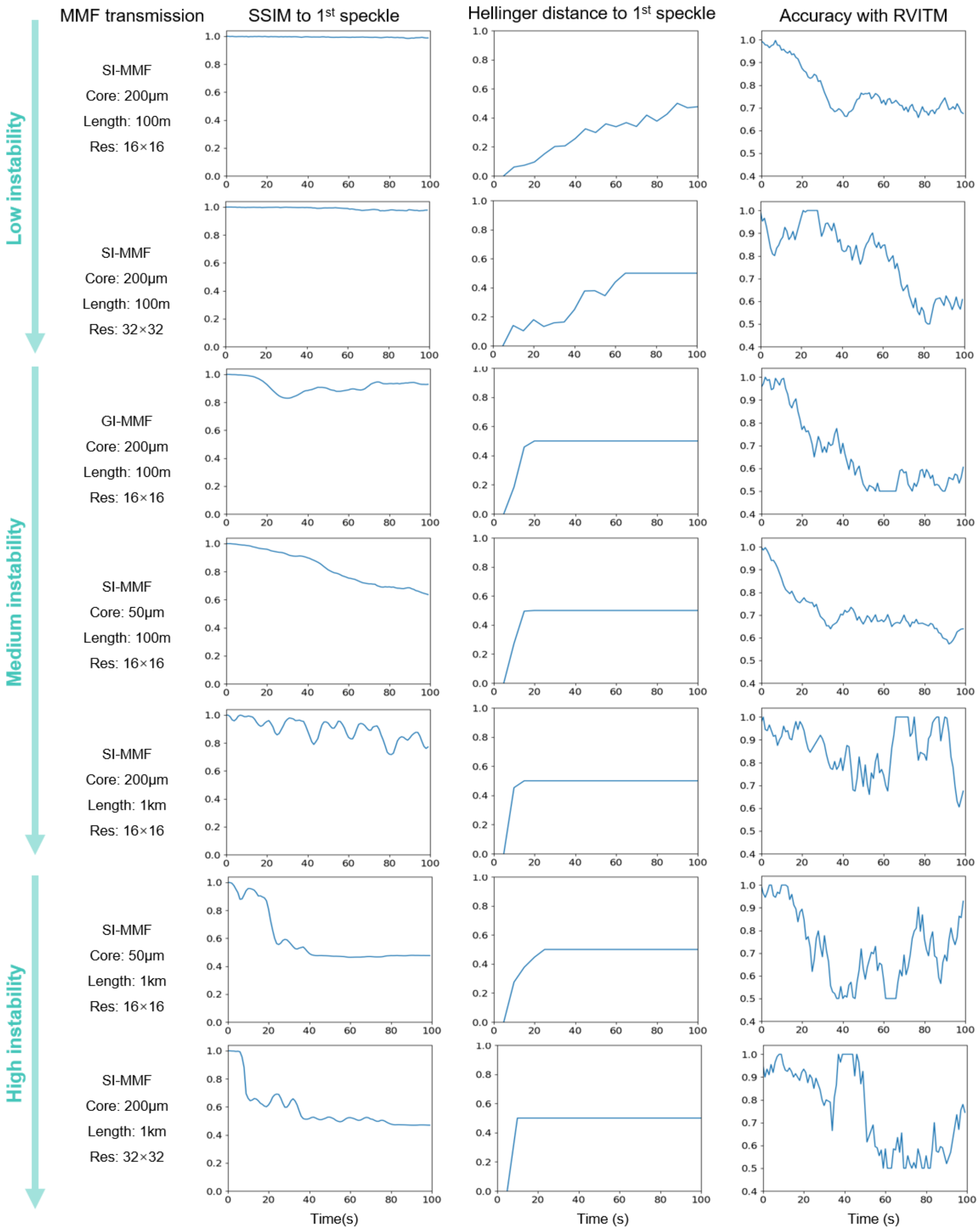

Supplementary Figure 2

Characterization of MMF transmission channel stability. We summarized the stability of seven representative MMF channels of varying configurations including core diameters of 50 μm and 200 μm, fiber lengths of 100m and 1km, fiber type of step-index (SI) and gradient-index (GI), and spatial resolution of 16x16-pixels and 32x32-pixels. We experimented for 100 seconds and calculated the metrics including SSIM and Hellinger distance between each output speckle image and the first one, as well as pixel-wise accuracy of the recovered input pattern using RVITM algorithm. The system instability rapidly grows as the fiber length increases or the core diameter reduces (from top to bottom).

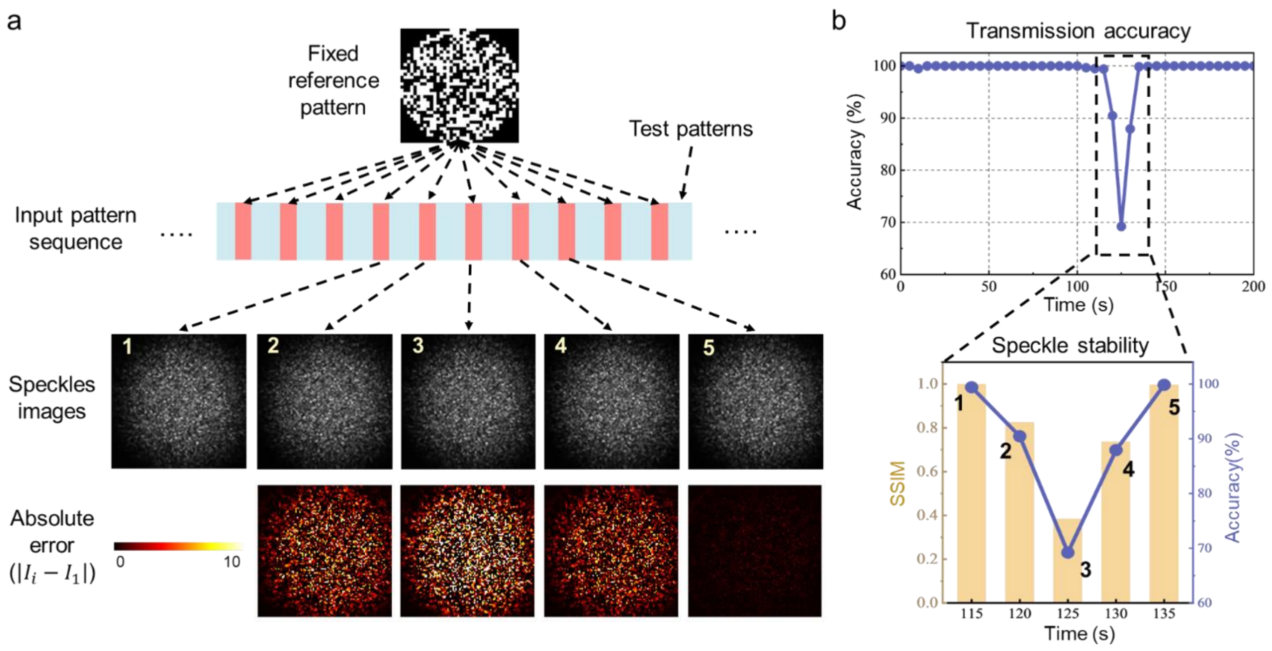

Supplementary Figure 3

Characterization of abrupt distortion of MMF channel. We repeatly inserted a fixed reference pattern at 5s-interval into a sequence of random test patterns for continuous spatial transmission through a 1km-long MMF. The reference pattern was used to evaluate the short-term stability and the test pattern sequence was used to calculate transmission accuracy. (a) Output speckle images of the reference pattern at five timepoints within the 115-135s were displayed. The intensity errors of timepoints 2~5 compared to timepoint 1 were presented, indicating an intense transmission model turbulence within the timepoint 2~4. (b) plots the accuracy during the whole transmission process, where up to 30% accuracy degradation was observed at the moment when abrupt distortion occurred. The SSIM analysis was further performed on the speckle images of reference pattern at timepoints 1-5.

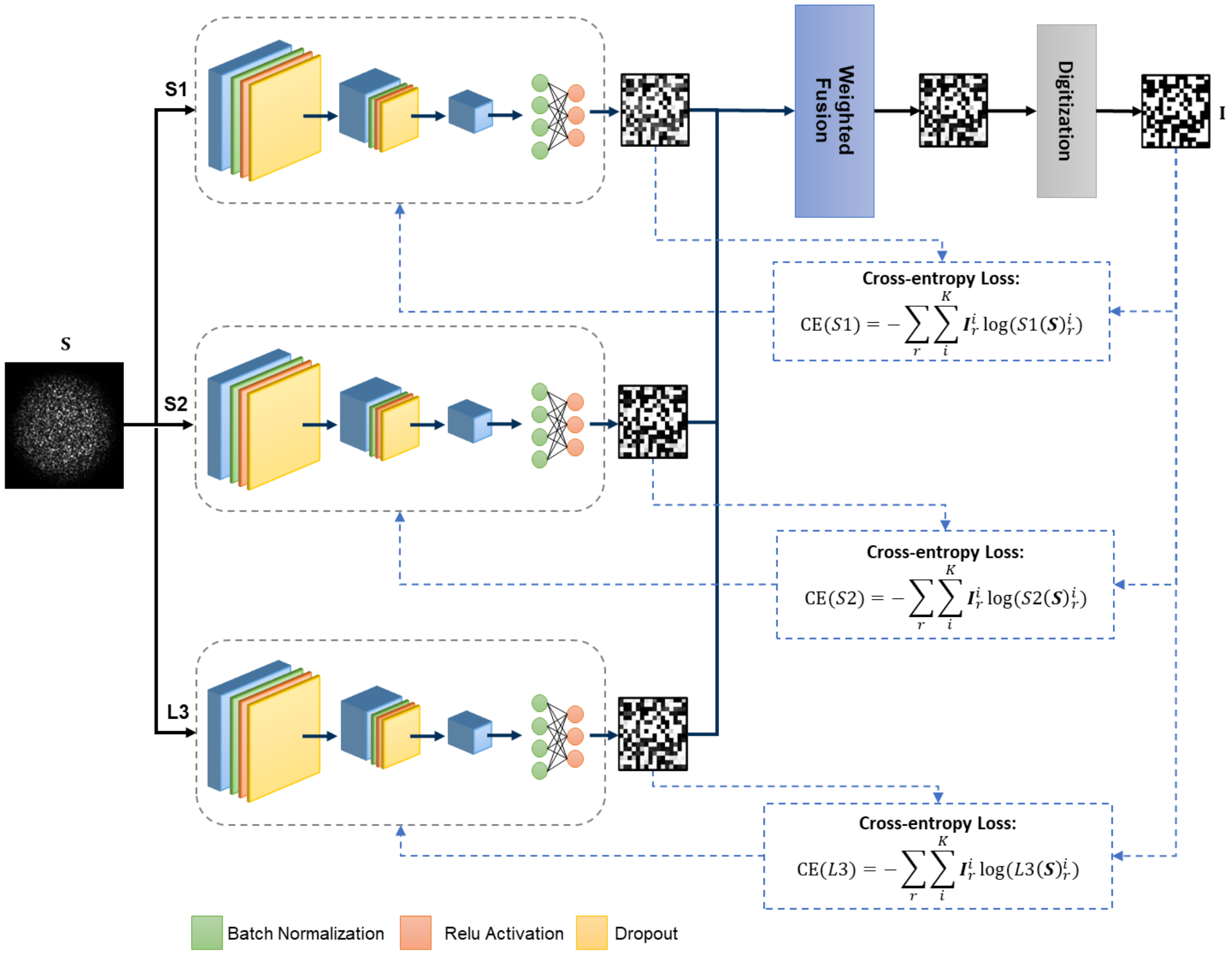

Supplementary Figure 4

Schematic of the multi-timescale network architecture and training. The ensemble network is composed of three sub-networks with the same structure, each consisting of two convolutional layers and one fully-connected layer. To regularize the network training, we leverage the Dropout to avoid over-fitting, and Batch Normalization to scale the feature after each convolution layer. The loss function for each sub-network in the MMDN framework is the cross-entropy loss between the sub-network prediction and the digitized ensemble prediction using the previous models. The cross-entropy loss between predicted and ground-truth images is calculated pixel-by-pixel and integrated as $\mathrm{CE}(f) = \sum_{r=1}^{N} - \sum_{i=0}^{K} \mathbf{I}_r^i \log\big(f(\mathbf{S})_r^i\big)$. Here, $\mathbf{S}$ is the input speckle pattern, the subnetwork prediction can be expressed as $f(\mathbf{S})$, $f = S1, S2, L3$, and the 'ground-truth' image is denoted as $\mathbf{I}$. The deviations come from two parts: 1) the adaptive fusion of three subnetworks gives an ensemble prediction that surpasses each sub-model; 2) the low-bit digitization acting like a nonlinear function helps to correct small errors.

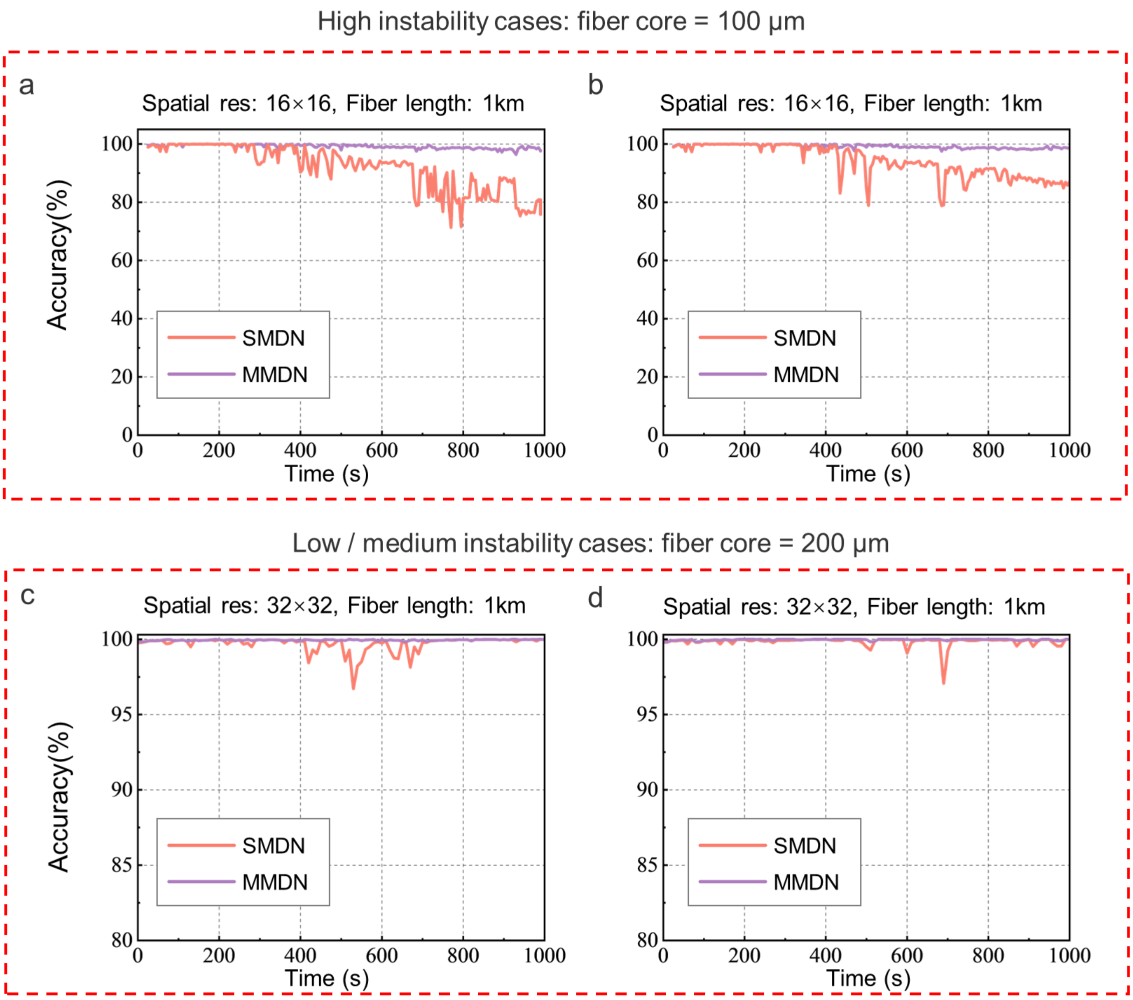

Supplementary Figure 5

Comparison of multi-scale memory ensemble and long-term only dynamic learning network. To evaluate the improvement in robustness brought by the Multi-scale Memory ensembling scheme of the proposed MMDN, we developed the Single-scale Memory Dynamic learning Network (SMDN) and compared their performance for spatial transmission in highly and mediumly unstable MMF channels. (a)(b) High unstability case of 16x16-pixel patterns transmission through 1km-length SI MMF with 100μm core diameter. (c)(d) Medium unstability case of 32x32-pixel transmission through 1km-length SI MMF with 200μm core diameter. In the highly unstable medium, traditional SMDN cannot adapt to the rapid changes in transmission characteristics, and the accumulated prediction error leads to an accuracy decrease after 200-300s duration. While the proposed MMDN can quickly adapt to system changes and hence shows superior transmission accuracy compared to SMDN. In medium stable transmission medium, we obverse a sharp accuracy decrease due to the abrupt periodic oscillation of the medium's transmission characteristics, while MMDN can adapt to these changes and significantly reduce the accuracy fluctuation. Comparative experiments indicate that compared to SMDN, multi-scale memory integration endows MMDN with higher robustness to support transmitting spatial information over a long period and with high precision through multimode fibers.

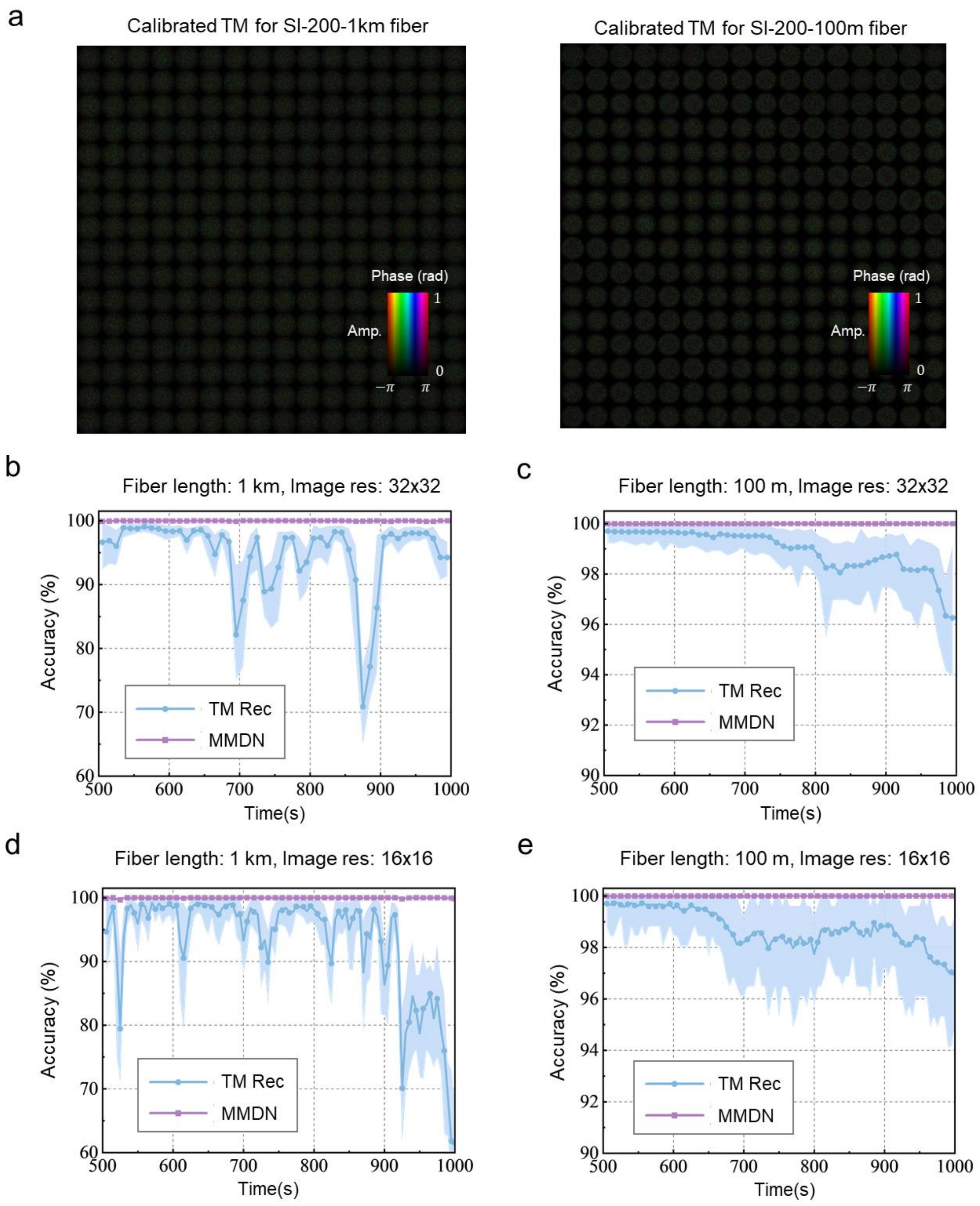

Supplementary Figure 6

Comparison in reconstruction accuracy between MMDN and TM-based algorithms. (a) Example of calibrated transmission matrix (TM) for 100m and 1km length MMFs (step-index, core dia. 200um, 0.22NA). The complex TM is measured with a phase retrieval-based approach using binary DMD for amplitude modulation[6]. The complex TM shown here is measured with 1024 pairs of 16x16-pixel input images and 100x100-pixel output speckles, while the TM for 32x32-pixel image recovery is measured with 4096 pairs of 32x32-pixel input images and 150x150-pixel output speckles. (b-e) Transmission accuracy for 500s duration experimented on various MMFs using proposed MMDN and TM-based reconstruction.

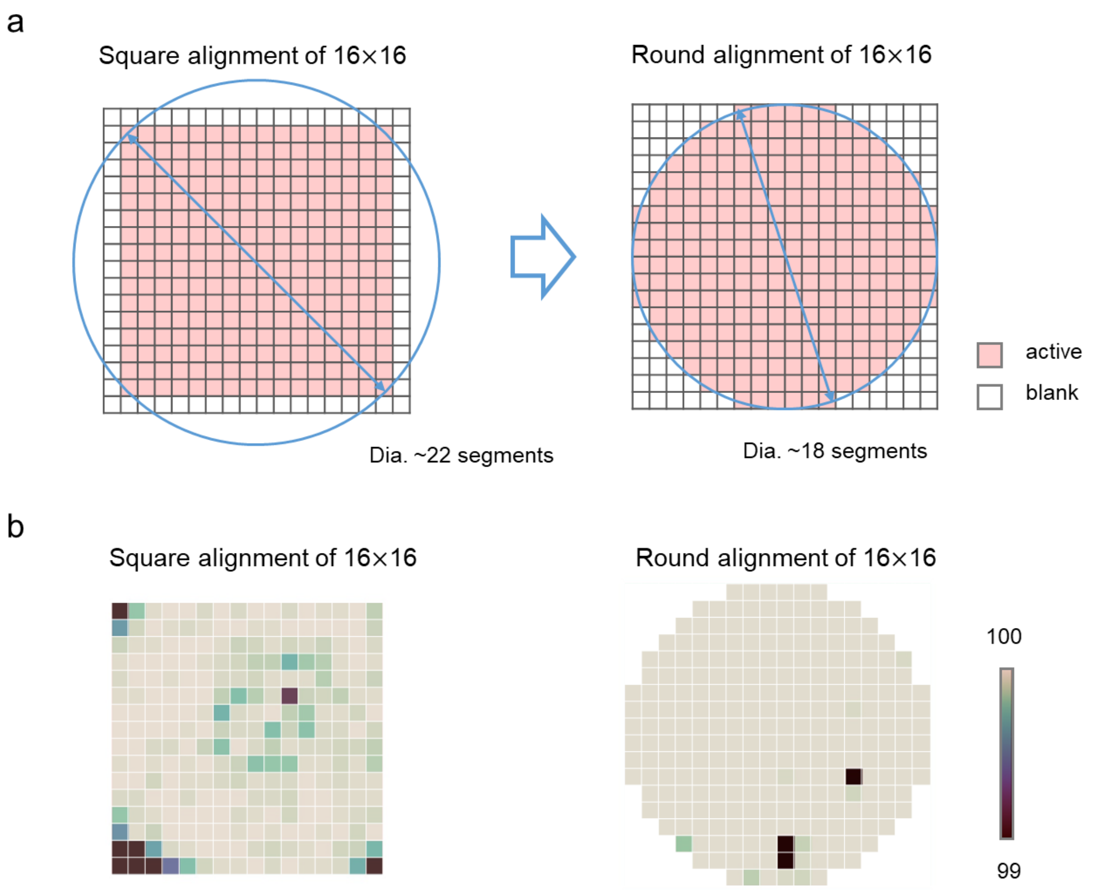

Supplementary Figure 7

Efficient pixel reassignment of the transmitted spatial patterns. (a) Concept of round distributed pixel assignment strategy. In previous works regarding 2D information transmission over MMF, the spatial information was multiplexed using a square assignment. However, we observed a significant decrease in transmission accuracy at the edges and corners of the 2D pattern in experimental tests. To make the transmitted 2D information better fit the shape of the fiber, we proposed a round assignment scheme for 2D patterns. (b) Experimental comparison of two coding formats. We tested the spatial transmission of 16x16-pixel patterns within a 1km MMF, and the spatial distribution of average both pixel assignment schemes was compared. We can see that the transmission accuracy of the round assignment scheme is significantly enhanced, especially at the corners.

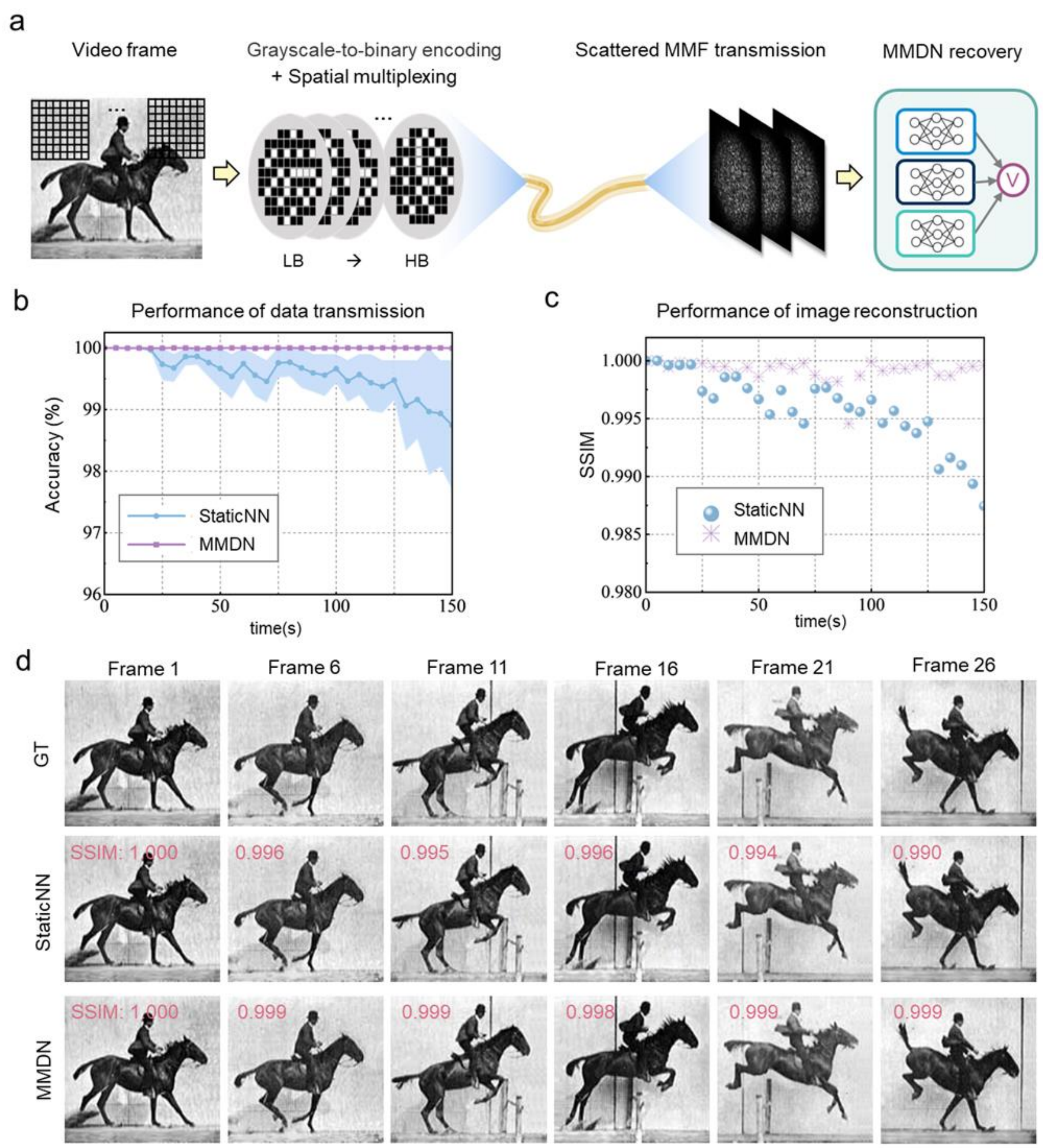

Supplementary Figure 8

Transmission of incompressive encoded video via MMFs. (a) Pipeline of incompressive encoded video transmission through spatial-multiplexed MMF channel and decoding with MMDN. To transmit the video frame of 8-bit precision and RGB format, we convert each pixel into a binary sequence of 24-bit length, and sequentially assemble the bit sequences into a set of spatial-multiplexed patterns. (b) Transmission accuracy of encoded signals. (c) SSIM of decoded video frames. (d) Example video frames recovered with StaticNN and MMDN. For the incompressive encoded video transmission scheme, the recovered image quality is much less vulnerable to the transmission accuracy decrease compared to the compressive scheme, yet the transmission throughput is lower (it takes about 150s to transmit the 31-frame video).

a

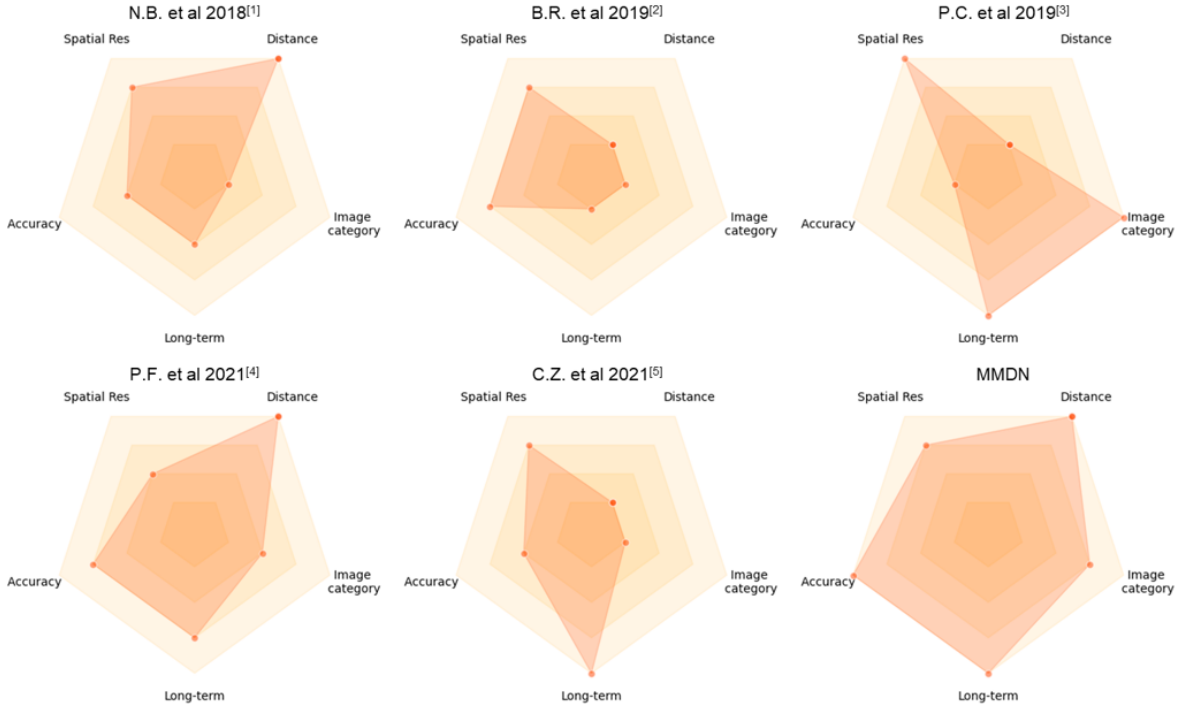

b

	N.B. et al 2018[1]	B.R. et al 2018[2]	P.C. et al 2019[3]	P.F. et al 2021[4]	C.Z. et al 2021[5]	MMDN
Distance [a]	1km	0.75m	1m	1km	1m	1km
Spatial resolution [b]	32x32	32x32	92x92	20x20	28x28	32x32
Accuracy [c]	ACC: 90.0%<br>/<br>/	/<br>PCC: ~0.93<br>/	/<br>PCC: 0.75~0.98<br>SSIM: 0.21~0.45	ACC: 94.9%<br>PCC: 0.92<br>SSIM: 0.932	/<br>/<br>SSIM: 0.65~0.775	ACC: > 99.9%<br>/<br>SSIM: > 0.995
Long-term capability	Not mentioned for image recovery; several hours for classification	Not mentioned	No image degradation for > 48hours	No image degradation for ≤ 200s	No image degradation for > 50hours	No image degradation for > 1000s
Image category	MNIST dataset	E-MNIST dataset	Arbitrary natural scene images	Arbitrary binary images	MNIST dataset	Arbitrary binary images

[a] maximum fiber length.
[b] maximum pixel number of input images.
[c] ACC: accuracy of binarized images; PCC: Pearson correlation coefficient; SSIM: structural similarity index measurement.

Supplementary Figure 9

Comparison of MMDN and state-of-art approaches for spatial decoding in MMFs. (a) Comparison of transmission distance, reconstruction accuracy, transmission duration, spatial resolution, and image category using previous reported approaches and proposed MMDN. (b) Specific performance parameters.

Specifications	Fig. 2a	Fig. 2b	Fig. 2c	Fig. 2d	Fig. 3a	Fig. 3b	Fig. 4 a,b	Fig. 4 c,d	Fig. 5-6
**Type**	Step-Index				Step-Index		Gradient-Index	Step-Index	Step-Index
**Core diameter**	200 µm				200 µm		200 µm	200 µm	200 µm
**Length**	1 km		100 m		1 km		100 m	1 km	1 km
**Image res**	32×32	16×16	32×32	16×16	24×24	16×16	16×16	32×32	32×32
**Grayscale**	1-bit				2-bit	4-bit	1-bit		1-bit

Specifications	Fig. S2							
**Type**	Step-Index	Step-Index	Gradient-Index	Step-Index	Step-Index	Step-Index	Step-Index	Step-Index
**Core diameter**	200 µm	200 µm	200 µm	50 µm	200 µm	50 µm	200 µm	200 µm
**Length**	100 m	100 m	100 m	100 m	1 km	1 km	1 km	1 km
**Image res**	16×16	32×32	16×16	16×16	16×16	16×16	32×32	32×32
**Grayscale**	1-bit							

Specifications	Fig. S3	Fig. S5 a,b	Fig. S5 c,d	Fig. S6b	Fig. S6c	Fig. S6d	Fig. S6e	Fig. S8
**Type**	Step-Index							
**Core diameter**	200 µm	100 µm	200 µm	200 µm				200 µm
**Length**	1 km	1 km		1 km	100 m	1 km	100 m	1 km
**Image res**	32×32	16×16	32×32	32×32		16×16		32×32
**Grayscale**	1-bit							

Supplementary Figure 10

Summary of the specifications of MMFs in all experiments. The fiber types, core diameter, length and image resolution and gray-level in all experiments are summarized in the table.

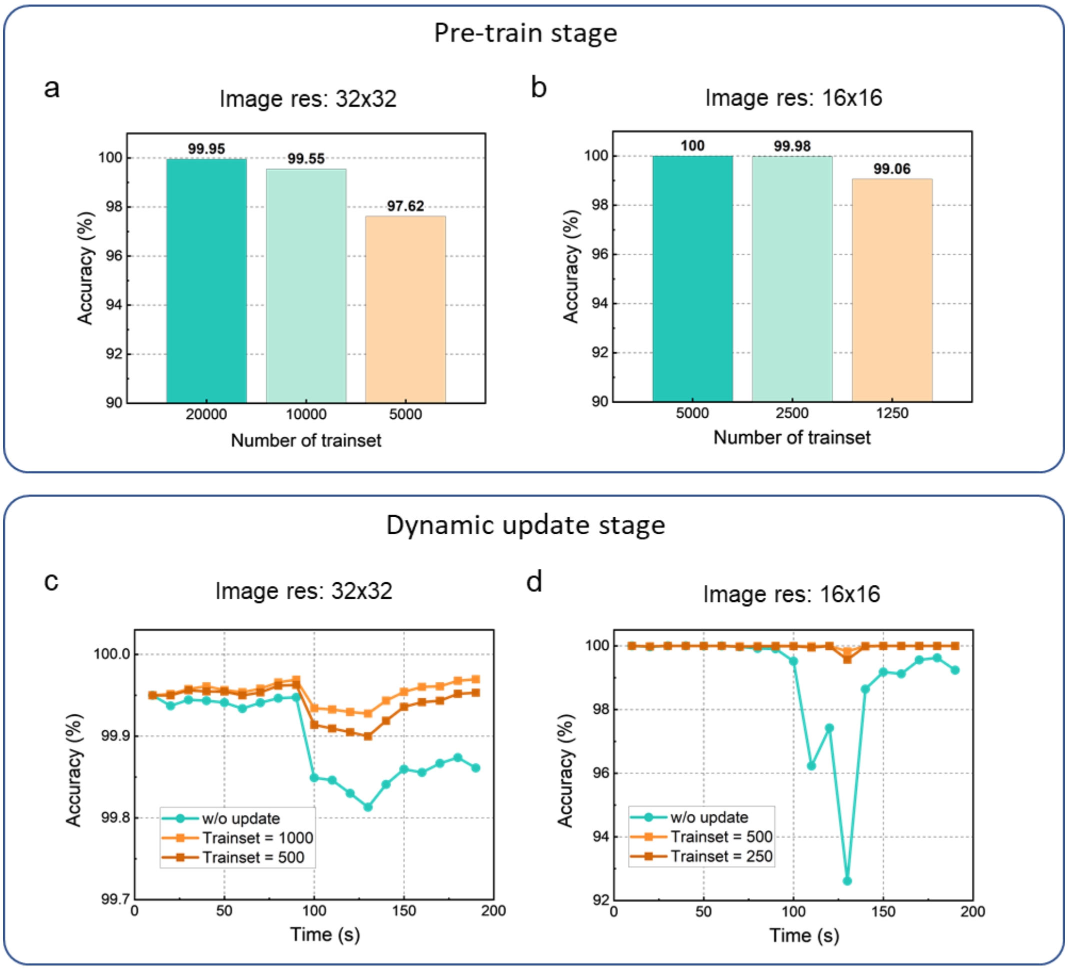

Supplementary Figure 11

Performance evaluation with respect to the number of training dataset. The network performance is evaluated when using different amount of training set. For the pre-training stage, we acquired 200-second 32×32-pixel data and randomly chose 20,000, 10,000, and 5,000 samples for training, and the accuracy on a validation set containing other 1000 samples is shown in (a). The validation accuracy for 16×16-pixel imaging training on 5,000, 2,500, and 1,250 samples is shown in (b). For each update stage, network update on a training set of 1000 and 500 paired data are tested for 32×32-pixel imaging, and the reconstruction accuracy in a 200s duration is shown in (c). For 16×16-pixel imaging, 500 and 250 paired data are tested for each update, as shown in (d). The reduction of data numbers will slightly reduce the accuracy at each update. Fiber parameters: Step-Index, 1 km length, core diameter 200 µm, NA 0.22.

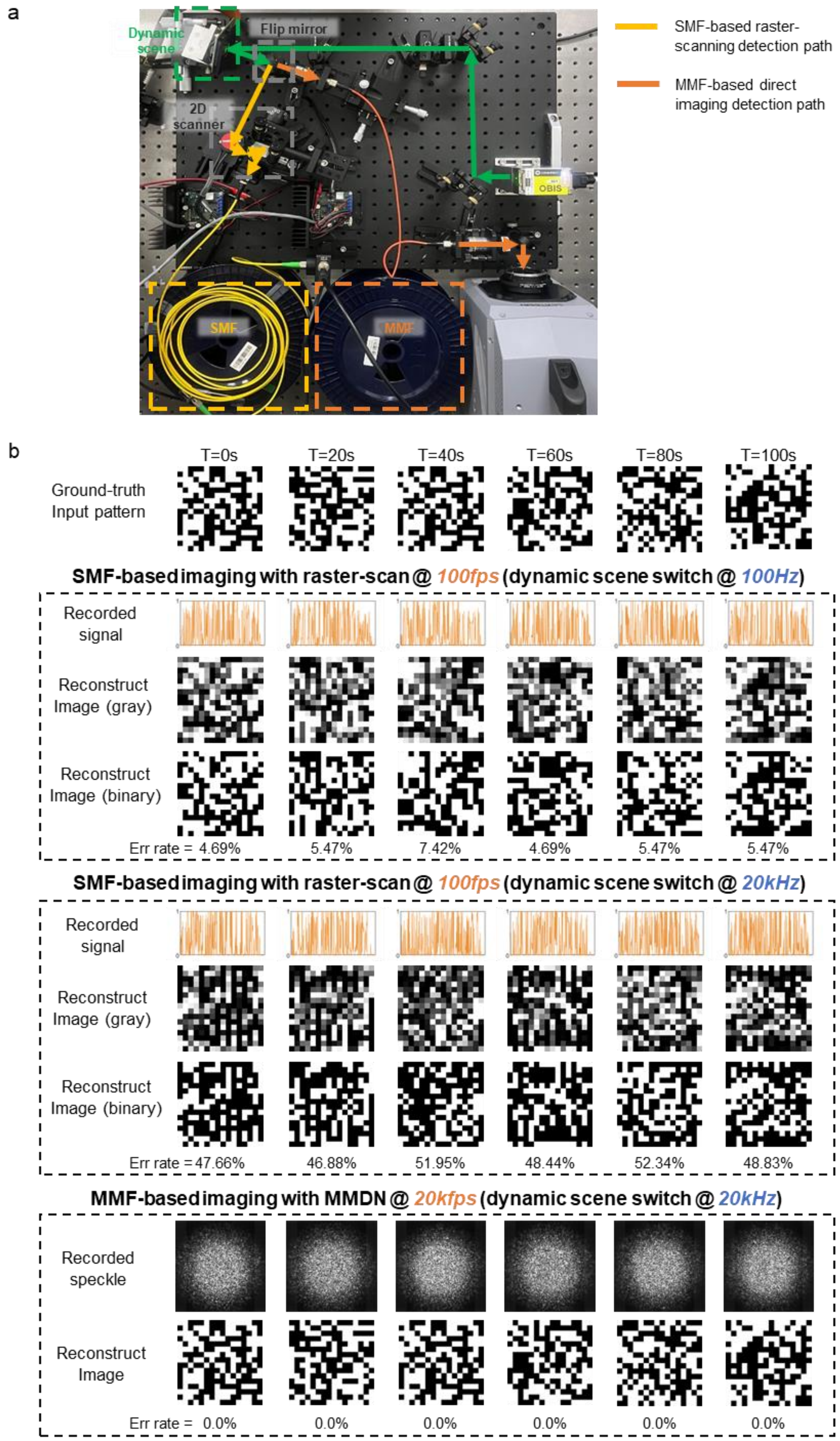

Supplementary Figure 12

Experimental comparison with SMF-based image transmission. (a) The optical setup of a SMF-and-MMF hybrid system. (b) Experimental reconstruction of SMF-based imaging with raster-scanning frame rate of 100fps and MMF-based imaging with 20k fps for dynamic scene recording.

Supplementary References:

[1] Borhani, Navid, et al. "Learning to see through multimode fibers." *Optica* 5.8 (2018): 960-966.

[2] Rahmani, Babak, et al. "Multimode optical fiber transmission with a deep learning network." *Light: science & applications* 7.1 (2018): 69.

[3] Caramazza, Piergiorgio, et al. "Transmission of natural scene images through a multimode fibre." *Nature communications* 10.1 (2019): 2029.

[4] Fan, Pengfei, et al. "Learning enabled continuous transmission of spatially distributed information through multimode fibers." *Laser & Photonics Reviews* 15.4 (2021): 2000348.

[5] Zhu, Changyan, et al. "Image reconstruction through a multimode fiber with a simple neural network architecture." *Scientific reports* 11.1 (2021): 1-10.

[6] Drémeau, Angélique, et al. "Reference-less measurement of the transmission matrix of a highly scattering material using a DMD and phase retrieval techniques." *Optics express* 23.9 (2015): 11898-11911.
