# Supplementary material for: Self-supervised dynamic learning for long-term high-fidelity image transmission through unstabilized diffusive media
Source: Nat Commun. 2024 Feb 19;15:1498. doi: 10.1038/s41467-024-45745-7 (PMC10876540; doi:10.1038/s41467-024-45745-7)
Supplement: Supplementary file 3 — Description of additional supplementary files [file 41467_2024_45745_MOESM3_ESM.docx]

**DESCRIPTION OF ADDITIONAL SUPPLEMENTARY FILES DOCUMENT**

**Supplementary Movie 1**

Demo of continuous transmission of generalized patterns via unstabilized long MMFs

**Supplementary Movie 2**

Demo of continuous transmission of hand-written letters and fashion images via 1km-length MMF

**Supplementary Movie 3**

Demo of high-throughput encoded video transmission via unstabilized 1kmlength MMF
