# Supplementary material for: Self-supervised dynamic learning for long-term high-fidelity image transmission through unstabilized diffusive media
Source: Nat Commun. 2024 Feb 19;15:1498. doi: 10.1038/s41467-024-45745-7 (PMC10876540; doi:10.1038/s41467-024-45745-7)
Supplement: Supplementary file 7 — Reporting Summary [file 41467_2024_45745_MOESM7_ESM.pdf]

nature portfolio

Corresponding author(s): Ziwei Li, Nan Chi, Qionghai Dai

Last updated by author(s): Dec 26, 2023

Reporting Summary

Nature Portfolio wishes to improve the reproducibility of the work that we publish. This form provides structure for consistency and transparency in reporting. For further information on Nature Portfolio policies, see our Editorial Policies and the Editorial Policy Checklist.

Statistics

For all statistical analyses, confirm that the following items are present in the figure legend, table legend, main text, or Methods section.

n/a	Confirmed	
☐	☒	The exact sample size (*n*) for each experimental group/condition, given as a discrete number and unit of measurement
☐	☒	A statement on whether measurements were taken from distinct samples or whether the same sample was measured repeatedly
☒	☐	The statistical test(s) used AND whether they are one- or two-sided<br>*Only common tests should be described solely by name; describe more complex techniques in the Methods section.*
☒	☐	A description of all covariates tested
☐	☒	A description of any assumptions or corrections, such as tests of normality and adjustment for multiple comparisons
☐	☒	A full description of the statistical parameters including central tendency (e.g. means) or other basic estimates (e.g. regression coefficient) AND variation (e.g. standard deviation) or associated estimates of uncertainty (e.g. confidence intervals)
☒	☐	For null hypothesis testing, the test statistic (e.g. *F*, *t*, *r*) with confidence intervals, effect sizes, degrees of freedom and *P* value noted<br>*Give P values as exact values whenever suitable.*
☒	☐	For Bayesian analysis, information on the choice of priors and Markov chain Monte Carlo settings
☒	☐	For hierarchical and complex designs, identification of the appropriate level for tests and full reporting of outcomes
☒	☐	Estimates of effect sizes (e.g. Cohen's *d*, Pearson's *r*), indicating how they were calculated

Our web collection on statistics for biologists contains articles on many of the points above.

Software and code

Policy information about availability of computer code

Data collection	The data is collected by a customized program.
Data analysis	The code for the reconstruction algorithm is provided with a GitHub link (https://github.com/fudanawei/MMDN.git).

For manuscripts utilizing custom algorithms or software that are central to the research but not yet described in published literature, software must be made available to editors and reviewers. We strongly encourage code deposition in a community repository (e.g. GitHub). See the Nature Portfolio guidelines for submitting code & software for further information.

Data

Policy information about availability of data

All manuscripts must include a data availability statement. This statement should provide the following information, where applicable:

- Accession codes, unique identifiers, or web links for publicly available datasets
- A description of any restrictions on data availability
- For clinical datasets or third party data, please ensure that the statement adheres to our policy

Source data are provided with this paper. One set of testing dataset to demonstrate the algorithm is provided in figshare with the identifier http://dx.doi.org/10.6084/m9.figshare.24249676.

Research involving human participants, their data, or biological material

Policy information about studies with human participants or human data. See also policy information about sex, gender (identity/presentation), and sexual orientation and race, ethnicity and racism.

Reporting on sex and gender	Not involved
Reporting on race, ethnicity, or other socially relevant groupings	Not involved
Population characteristics	Not involved
Recruitment	Not involved
Ethics oversight	Not involved

Note that full information on the approval of the study protocol must also be provided in the manuscript.

Field-specific reporting

Please select the one below that is the best fit for your research. If you are not sure, read the appropriate sections before making your selection.

☒ Life sciences ☐ Behavioural & social sciences ☐ Ecological, evolutionary & environmental sciences

For a reference copy of the document with all sections, see nature.com/documents/nr-reporting-summary-flat.pdf

Life sciences study design

All studies must disclose on these points even when the disclosure is negative.

Sample size	In our image transmission experiments, about 100~1000s acquisition providing 10k~100k samples are tested. This is a relative large quantity of testing samples to prove the performance of our proposed reconstruction technique.
Data exclusions	No data were excluded.
Replication	Five duplicated experiments were conducted for the 200um diameter step-index MMF transmission and all replicates succeeded. The other experiments conducted under different MMF configurations and on different testing datasets were conducted once.
Randomization	The patterns for testing are randomly generated or randomly selected from the original standard datasets.
Blinding	Blinding was not relevant to our study. All experimental procedures and data analyses were conducted based on objective, pre-defined standards. The experimental design did not involve subjective judgment regarding the treatment or outcomes, therefore, blinding was not applicable or necessary.

Reporting for specific materials, systems and methods

We require information from authors about some types of materials, experimental systems and methods used in many studies. Here, indicate whether each material, system or method listed is relevant to your study. If you are not sure if a list item applies to your research, read the appropriate section before selecting a response.

Materials & experimental systems

n/a	Involved in the study	
☒	☐	Antibodies
☒	☐	Eukaryotic cell lines
☒	☐	Palaeontology and archaeology
☒	☐	Animals and other organisms
☒	☐	Clinical data
☒	☐	Dual use research of concern
☒	☐	Plants

Methods

n/a	Involved in the study	
☒	☐	ChIP-seq
☒	☐	Flow cytometry
☒	☐	MRI-based neuroimaging

Plants

Seed stocks	Not involved
Novel plant genotypes	Not involved
Authentication	Not involved
